# 

**Published:** 1885-03

**Authors:** 


					﻿OLD SERIES
Vol. XXV .
NEW SERIES
Z?V /	Vol. II—No. I.
k	2
WiSS
fflEDIGAL^^URiS
> jOURNAESB
tAeri. at-—— -----------Lw>
1 r I11 RY W.F. WESTMORELAND, ,J
UjA H.V.M.MII±ER,M.D.LL.D.jKi \
JAMES A.GRAY, M.D.^W
rflBLlSHED BY JAMES P.HARRISON&.Cffl
y_________________Atlanta ,ga. aS
SUBSCRIPTION: 2.LO A YEAR.
				

